# Design and methods of the Ixekizumab Diabetes Intervention Trial (I-DIT): protocol for a phase 2, randomised, multicentre, placebo-controlled, double-blind trial of anti-interleukin 17 as a treatment option for adults with new-onset type 1 diabetes

**DOI:** 10.1136/bmjopen-2025-103486

**Published:** 2025-11-12

**Authors:** Shilan Seyed Ahmadi, Olle Korsgren, Per-Anders Jansson, Marcus Lind

**Affiliations:** 1 Department of Molecular and Clinical Medicine, Institute of Medicine, Sahlgrenska Academy, University of Gothenburg, Gothenburg, Sweden; 2 Department of Medicine, Geriatrics and Emergency Care, Sahlgrenska University Hospital, Gothenburg, Västra Götaland, Sweden; 3 Department of Immunology, Genetics and Pathology, Uppsala University, Uppsala, Sweden; 4 Department of Medicine, NU Hospital Group, Trollhättan and Uddevalla, Sweden

**Keywords:** IMMUNOLOGY, DIABETES & ENDOCRINOLOGY, Clinical Protocols

## Abstract

**Introduction:**

Type 1 diabetes is characterised by progressive loss of pancreatic beta cells. Studies have shown that interleukin (IL)−17 is likely a mediator for this destruction. Whether inhibition of IL-17 could preserve beta cell function in people with new-onset type 1 diabetes is unknown.

**Methods and analysis:**

In this phase 2, randomised, multicentre, placebo-controlled, double-blind trial conducted at 17 study sites in Sweden, 127 adults aged 18–45 years old with newly diagnosed type 1 diabetes will be enrolled. Participants will be randomised to receive either subcutaneous IL-17 inhibitor or placebo for 52 weeks, in addition to their conventional therapy. The primary endpoint will be change in residual insulin secretion measured by the area under the curve for C-peptide in response to 2-hour mixed meal tolerance test between baseline and week 52. Additionally, masked continuous glucose monitoring will be performed during 14 days at the run-in period, week 13, week 26 and week 52. Secondary endpoints will be change in time in glucose range (3.9–10 mmol/L), time in hypoglycaemia (<3.9 mmol/L), HbA1c and mean insulin dosage per kilogram body weight. Patient-reported outcomes will be evaluated with questionnaires at baseline, week 26 and 52. Additionally, 1 and 3 years after the end of the treatment period, the participants will be examined during a visit regarding endogenous insulin production, glycaemic control, glucose metrics and insulin doses.

**Ethics and dissemination:**

Approvals were obtained from the Swedish Ethical Review Authority (Dnr 2020–05098) and the Swedish Medical Products Agency (Dnr 5.1-2021-105808) before participant enrolment. Participants provide informed consent before inclusion. Results of this study will be submitted for publication in international peer-reviewed journals and key findings will be presented at international scientific conferences.

**Trial registration number:**

ClinicalTrials.gov, NCT04589325.

**STRENGTHS AND LIMITATIONS OF THIS STUDY**

- This is a randomised, double-blind, placebo-controlled trial that examines the effect of interleukin-17 inhibition on endogenous insulin secretion in people with newly diagnosed type 1 diabetes.
- Secondary outcomes of the study will offer information regarding insulin doses and glucose metrics measured by masked continuous glucose monitoring.
- The extension phase will give further knowledge whether a potential beneficial effect is sustained over time.
- A limitation is that there will be no data on how children respond to the treatment.
- Another limitation is the relatively short follow-up period.

## Introduction

Type 1 diabetes is characterised by a destruction of insulin-producing beta cells in the pancreas.^1^ The development of type 1 diabetes occurs in three stages: In stage 1, asymptomatic individuals have the presence of >2 pancreatic autoantibodies; in stage 2, impaired glucose tolerance is seen; and in stage 3, type 1 diabetes eventually develops.^2^ Thereafter, in most individuals, there is a gradual loss of the remaining insulin-producing cells after diagnosis over a period of several years, or even decades.^3^,^5^ These long-lasting beta cells remain functional decades after diagnosis, as evidenced by increased secretion of C-peptide in response to a mixed meal.^6^

Already in the beginning of the year 2000, several studies suggested that interleukin (IL)-17 plays a key role in the destruction of beta cells and the development of type 1 diabetes.^7^,^15^ In the Ixekizumab Diabetes Intervention Trial (I-DIT), we aim to evaluate if endogenous insulin secretion can be preserved or increased in people with newly diagnosed type 1 diabetes by inhibition of IL-17 compared with placebo in addition to their conventional therapy. We will also evaluate change in glucose metrics measured by masked continuous glucose monitoring (CGM), HbA1c, mean insulin doses per kilogram body weight and various patient-reported outcomes.

## Methods and analysis

### Study design and setting

**Figure 1:**
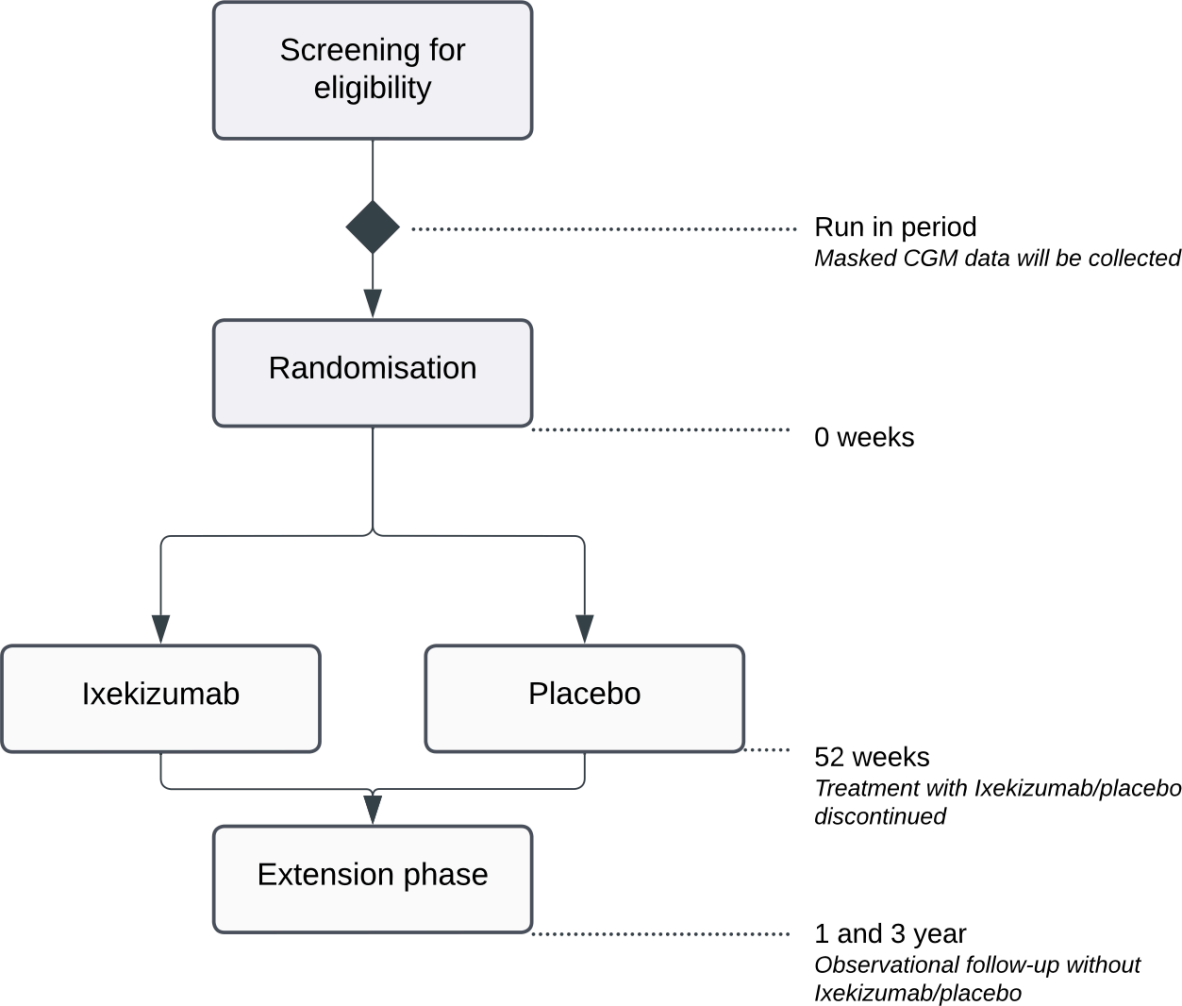
Study design flowchart.

The present study will be an investigator-initiated, multicentre, double-blind, placebo-controlled, randomised trial including 127 newly diagnosed individuals with type 1 diabetes from 17 study sites in Sweden (online supplemental material). After a run-in period of 2 weeks where blinded CGM is worn to collect baseline variables, study participants will be followed for 12 months after randomisation. Additionally, 1 and 3 years after the end of the treatment period, the participants will be examined during a visit regarding insulin production, glycaemic control, glucose metrics and insulin doses (figure 1). The trial is investigator-initiated and sponsored by the University of Gothenburg, Gothenburg, Sweden. The trial started including study participants in September 2022. The last patient is anticipated to be randomised in May 2025.

### Screening

Individuals with type 1 diabetes are monitored at their own clinic and receive verbal or written information about the study during a visit at the hospital, or written information via mail and verbal information via telephone. If individuals are interested in participating, verbal and written informed consent will be obtained from each individual by an investigator (physician) at the study site. During the screening visit, inclusion and exclusion criteria will be examined. Major inclusion criteria are diagnosis of type 1 diabetes and first injection of insulin within 100 days prior to screening, age 18–45 years old, presence of at least one of the autoantibodies (insulin/IAA, GAD-65, ZnT8 or IA-2) at clinical practice or at screening and have a remaining stimulated peak C-peptide >0.20 nmol/L during a mixed meal tolerance test (MMTT). For all inclusion and exclusion criteria, see table 1.

**Table 1:** Inclusion and exclusion criteria

Inclusion criteria	Exclusion criteria
Subjects eligible for inclusion in the study have to fulfil all of the following criteria: Signed informed consent and expected cooperation of the patients for the treatment and follow-up must be obtained and documented according to ICH GCP, and national/local regulations before trial activities are begun. Must be willing and capable of taking the study drugs and meet for tests and follow-up as described. Diagnosed type 1 diabetes (E10.9) within 100 days. First injection of insulin maximum 100 days prior to screening. If aged 36–45 years, a current insulin regimen of both basal and prandial insulin or alternatively use of an insulin pump should exist. Aged 18–45 years old. Presence of antibodies at clinical practice or at screening to at least one of the following antigens: insulin/IAA, GAD-65, IA-2 and ZnT8. Remaining stimulated peak C–peptide ≥0.20 nmol/L. If age 36–45 years, peak C-peptide should be <2.0 nmol/L. Effective contraceptive methods throughout the study.	Subjects fulfilling any of the following criteria are not eligible for inclusion in this study: Contraindications to ixekizumab. Treatment with any oral or injected glucose-lowering agents other than insulin. A history of haemolytic anaemia or significantly abnormal haematology/coagulation results at screening. Participation in other clinical trials with a new chemical entity within the previous 3 months. Subjects with severe obesity (BMI >35 kg/m^2^ if age 18–35 years and BMI >30 kg/m^2^ if age 36–45 years). Subjects with other autoimmune diseases, for example, Mb Crohn, Ulcerative colitis, Graves' disease, psoriasis, psoriatic arthritis and axial spondylarthritis, except coeliac disease and hypothyroidism, which do not need to be excluded. Active serious or chronic infections (ie, in case the patient had a serious infection (eg, pneumonia, cellulitis), has been hospitalised, has received intravenous antibiotics for an infection within 12 weeks prior to screening visit, had a serious bone or joint infection within 24 weeks before screening visit, has ever had an infection of an artificial joint. Known immunodeficiency or patient is immunocompromised to an extent that participation in the study would pose an unacceptable risk to the patient. Active or latent tuberculosis. HIV or active hepatitis B or C. Active or recurrent fungal infection. Subjects with myocardial infarction, stroke, unstable angina or heart failure in the last 6 months. Current clinically significant cardiac arrhythmias as verified by ECG. Planned surgery during the treatment period of the study (except minor surgery on skin lesions, eg, nevus). For female subjects: Positive pregnancy test, presently breast-feeding or unwillingness to use effective contraceptive measures for the duration of the study and 3 months after discontinuation. For male subjects: intent to procreate during the duration of the study or within 3 months after discontinuation or unwillingness of their partner to use effective contraceptive measures for the duration of the study and 4 months after discontinuation. Any history of malignancy in the last 5 years except for completely resected squamous or basal cell carcinoma of the skin. Administration of live attenuated vaccine(s) (LAV) within 2 months of enrolment. Or intended use of LAV during the treatment period. The investigator judges that the clinical diagnosis of type 1 diabetes set is incorrect or uncertain (needs to be confirmed by discussion with experienced diabetologist if excluding due to this criterion) Allergy against ingredients of the investigational products. Known allergy or hypersensitivity to any biologic therapy (active substance or excipients) that would pose an unacceptable risk to the patient if participating in the study. Presence of serious disease or condition, which in the opinion of the investigator makes the patient non-eligible for the study. Liver injury criteria: patients with active hepatobiliary diseases or at screening having alanine aminotransferase (ALT) or aspartate aminotransferase (AST) >2.5 times the upper limit of normal (>2.5 x ULN). Laboratory abnormalities at screening: Neutrophil count <1500 cells/μL (=1,5 *10^9^ cells/ L) Platelet count <100 000 cells/μL (= 100 *10^9^ cells/ L) Haemoglobin <8.5 g/dL (= <85 g/L) (males) and <8 g/dL (= <80 g/L) (women)

* For details regarding contraceptive methods, see online supplemental materials.

### Randomisation

If inclusion criteria are fulfilled, randomisation will take place no later than 8 weeks later. The participants will be randomised to either treatment with an IL-17 inhibitor (ixekizumab) or placebo.

A randomisation list will be created for each participating site. The lists are created by Statistiska Konsultgruppen, Gothenburg, and sent to Tamro AB, Gothenburg, which is responsible for delivering the study medications to each participating site. Only designated personnel from Statistiska Konsultgruppen and Tamro AB will have access to the randomisation lists. If a participant discontinues study participation, their randomisation number will not be reused, and the subject will not be allowed to re-enter the study again.

### Dose and administration

Ixekizumab/placebo will be administered by the participant via subcutaneous (sc.) injections for a total treatment period of 12 months. Two sc. injections (160 mg) will be administered at week 0, one dose (80 mg) at week 2, 4, 6, 8, 10 and 12 and continue with a maintenance dose (80 mg) every fourth week for a total treatment period of 12 months. The same dosing schedule is used for treatment of psoriasis.

The syringes for ixekizumab and placebo will have a similar look and not be possible to distinguish. Each syringe will contain 1 mL solution of ixekizumab or placebo. The syringes containing ixekizumab will deliver 80 mg of ixekizumab.

Ixekizumab and placebo will be provided by Eli Lilly and Company through their investigator-initiated trials program.

### Concomitant medication

Concomitant medications and other treatments known to influence glucose levels prescribed before study entry will, if possible, remain unchanged during the study period unless changes are made for safety reasons. This includes types of insulins, as well as glucose sensors and types of insulin delivery (injections/insulin pump). Concomitant medication will be recorded in the electronic Case Report Form (eCRF) at every clinical visit.

### Adherence to treatment

Compliance and adherence to protocol and treatment will be checked at each contact with the participant. The unused amount of trial product will be assessed against the dispensed amount, and, in case of discrepancies, the participant must be asked about compliance.

### Continuous glucose monitoring

Masked CGM will be performed during 14 days at each of the following time points: run-in period, week 13, week 26 and week 52. At week 52, masked CGM will be set at some time point during the 4 weeks preceding the 52 week visit since masked CGM needs to be collected during treatment with ixekizumab/placebo. The participant’s own CGM use for their conventional treatment should be continued and changes will be determined by regular clinical diabetes care.

### Study visits

The visit schedule will be as follows: randomisation, week 2, 4, 13, 26, 39 and 52. MMTT will be performed at randomisation, week 4, 26 and 52. At all visits, physical examinations will be performed and HbA1c collected. Blood pressure and weight will be taken at all visits except week 2. A detailed description of the trial procedures is provided in table 2. Additionally, participants will complete a series of questionnaires evaluating intervention treatment satisfaction, well-being, hypoglycaemia confidence, diabetes-related distress and physical activity at the study site:

- Diabetes Treatment Satisfaction Questionnaire (DTSQ) is an 8-item scale measuring aspects of satisfaction scored on a 7-point Likert scale, including domains of current treatment satisfaction, convenience of treatment, understanding of diabetes, recommendation of treatment and continuation of treatment. Two versions will be used: the DTSQs and DTSQc. DTSQs is used to record current treatment satisfaction throughout the trial and the DTSQc is used to retrospectively compare the treatment arms at week 52.^16^

**Table 2:** Trial procedures

	Patient info (two visits)	Screening visit (fasting*)	Baseline randomisation (fasting)	Week 2 Follow-up±1 week	Week 4 Follow-up±10 days (fasting)	Week 13 Follow-up±2 weeks	Week 26 Follow-up±2 weeks (fasting)	Week 39 Follow-up±2 weeks	Week 52 Follow-up±2 weeks (fasting)		Weeks 104 and 208 Extension phase (±3 months) (fasting)	
Variables	Visit 1	Visit 2	Visit 3	Visit 4	Visit 5	Visit 6	Visit 7	Visit 8	Visit 9		Visit 10+11	
Information visit	X											
Obtain informed consent	X											
Inclusion/exclusion criteria		X										
ECG		X										
Demographics		X										
Length		X										
Medical history		X	X						X			
Date of diagnosis of type 1 diabetes and first insulin injection		X										
Smoking status			X									
Family history of type 1 diabetes		X										
Concomitant medication		X	X	X	X	X	X	X	X			
Physical examination		X	X	X	X	X	X	X	X			
Blood pressure			X		X	X	X	X	X			
Weight		X	X		X	X	X	X	X			
Information regarding type of glucose monitoring			X			X	X	X	X		X	
GAD, ZnT8, insulin/IAA and IA-2-antibodies		X							X			
Mixed Meal Tolerance Test		X	X		X		X		X		X	
HbA1c		X	X		X	X	X	X	X		X	
Proinsulin			X						X			
Hematology†		X	X		X		X		X			
Clinical Chemistry‡		X	X		X		X		X			
Urine pregnancy test		X										
HIV, hepatitis serology, IgA transglutaminase, thyroid status		X										
Tuberculosis screening (QuantiFERON and chest X-ray)		X										
Liver status§		X	X		X		X		X			
Downloading CGM data from Libre Pro iQ		*	X			X	X		**	X	***	X (2 visits)
Sampling for biobank			X		X		X		X			
Types of insulin and doses of insulin			X		X		X		X		X	
Questionnaries¶			X				X		X			
Adverse events			X	X	X	X	X	X	X			

*During this visit, Libre Pro iQ will be initiated to collect masked CGM data.

**CGM will be initiated 4 weeks preceding this visit and the data collected at week 52.

***CGM will be initiated at week 104 and 208 and the data will be collected at a separate visit.

* Blood samples will be taken in a fasting state.

† Hb, MCV, WBC, platelets, absolute counts of neutrophils (segmented), lymphocytes, monocytes, eosinophils, basophils.

‡ Na, K, Ca, CRP, Creatinine.

§ 3AST, ALT, Alkaline phosphatase, Bilirubin (total and direct).

¶ DTSQs, WHO-5, SWE-HCS, SWE-PAID-20 and IPAQ (at Week 52, DTSQc is included).

- World Health Organization-5 (WHO-5) is a 5-item scale that assesses patient well-being.^17^
- The hypoglycaemia confidence questionnaire is a 9-item scale that evaluates patient confidence regarding their ability to prevent and address hypoglycaemic events.^18^
- Problem Areas in Diabetes Scale, Swedish version (SWE-PAID) is a 20-item scale which assesses worries and concerns specifically related to diabetes and its management. It has been shown to be a good marker of diabetes-related distress.^19^
- International Physical Activity Questionnaire (IPAQ) is a 7-item scale that assesses minutes spent in light, moderate and vigorous physical activity per week. Scores are expressed as energy expenditure in estimated metabolic equivalent task minutes.^20^

### Mixed meal tolerance test

The golden standard for evaluating endogenous insulin production is a 2-hour MMTT.^21^ The participants will consume a standard liquid meal (Fresubin) after an overnight fast >10 hours before the test. The liquid consists of fat, carbohydrates and protein based on body weight. P-glucose and c-peptide will be measured with seven sampling time points (−10, 0, 15, 30, 60, 90 and 120 min) after consuming the liquid. For details, see the procedure in the online supplemental material.

### Dropouts

Participants who drop out from the study will be asked for a follow-up on an additional visit and the following blood samples will be checked: HbA1c, C-peptide and proinsulin. Insulin doses and concomitant medications will be recorded. The dropouts will not be replaced.

### Post-study treatment

There will be no difference from the expected normal treatment of type 1 diabetes after the participants have ended the participation in the trial.

### Endpoints

The primary endpoint will be the change in residual insulin secretion measured by stimulated C-peptide 2-hour area under the curve profile measured by MMTT between baseline and week 52. The secondary endpoints are insulin need (U/kg), time in glucose range (3.9–10 mmol/L), time in hypoglycaemia (<3.9 mmol/L) and HbA1c. Exploratory variables include glycaemic variability, well-being, hypoglycaemia confidence, treatment satisfaction and diabetes-related distress. All predefined endpoints are shown in box 1.

Box 1 **Predefined endpoints**

**Primary endpoint**

Change in residual insulin secretion measured by stimulated C-peptide 2 hour area under the curve profile measured by mixed meal tolerance test (MMTT) between baseline and week 52.

**Secondary endpoints**

1. Change in mean Insulin dosage per kilo body weight for 24 hours from baseline to week 52.
2. Change in time with glucose levels in range (3.9-10 mmol/l) measured by masked CGM (Libre Pro iQ) from baseline to week 52.
3. Change in time with hypoglycaemia (<3.9 mmol/l) measured by masked CGM (Libre Pro iQ) from baseline to week 52.
4. Difference in HbA1c from baseline to week 52.

**Exploratory endpoints**

1. Change in time in hypoglycaemia (<3.0 mmol/l) measured by masked CGM (Libre Pro iQ) from baseline to week 52.
2. Change in proinsulin/c-peptide ratio in serum as a measure of beta cell stress from baseline to week 52.
3. Change in time in target (3.9-8 mmol/l) measured by masked CGM (Libre Pro iQ) from baseline to week 52.
4. Change in time in hyperglycaemia >10 mmol/l and >13.9 mmol/l measured by masked CGM (Libre Pro iQ) from baseline to week 52.
5. Change in glycaemic variability measured by SD, CV and MAGE by masked CGM (Libre Pro iQ) from baseline to week 52.
6. Change in proportion of patients with peak residual insulin secretion measured by MMTT: stimulated C-peptide >0.4 nmol/mL from baseline to week 52.
7. Change in WHO-5 scores from baseline to week 52.
8. Change in DTSQs scores from baseline to week 52.
9. Change in DTSQc scores estimated at week 52.
10. Change in HCS scores from baseline to week 52.
11. Change in PAID scores from baseline to week 52.
12. Change in IPAQ scores from baseline to week 52.

The same variables described above regarding primary, secondary and exploratory endpoints will be evaluated if the variable is measured at a specific time point from baseline to week 4, baseline to week 13 as well as baseline to week 26.

### Adverse events and safety

All adverse events (AE) will be collected. The recording and follow-up of AEs will be made from the time when study treatment is started until the end of study treatment. Serious AEs will be followed up until resolved or the event stabilises.

### Lab, monitoring and study drug delivery

The majority of blood samples, including C-peptide levels measured during the MMTT as for the primary endpoint, will be analysed at a central laboratory at the Karolinska University Hospital Laboratory. In brief, centrally analysed blood samples include islet cell autoantibodies, safety blood samples (including renal and liver function), viruses (HIV and hepatitis), thyroid function and coeliac biomarkers.

Only blood samples that require rapid analysis due to transport sensitivity will be handled locally; this includes glycated haemoglobin (HbA1c). The effect on HbA1c is a secondary endpoint and, therefore, must be standardised as much as possible. To ensure consistency, the same laboratory method will be used. In Sweden, all HbA1c methods are calibrated against a central standardisation system via EQUALIS, ensuring comparable HbA1c levels. At the local site, a chest X-ray will also be performed to screen for active/latent tuberculosis.

Biobank samples in whole blood, serum and plasma for all participants will be collected, and in a subsample of participants, also peripheral mononuclear cells.

Each study site will be monitored before the start of the trial, repeatedly during the study and at the end of the trial. Gothia forum, Gothenburg, Sweden, will monitor the trial. No data monitoring committee is used in this study since it is relatively small, short in duration and involves a medication already approved for other indications (eg, psoriasis).

Eli Lilly and Company provides study medication and Tamro AB handles study treatment logistics.

### Statistical analysis

The full analysis set (FAS-population) consists of all participants who receive at least one dose of study medication and have at least one follow-up measurement.

The per-protocol population (PP-population) consists of all participants in the full analyses set without any significant protocol deviations. The PP-population is defined at the clean file meeting before the database is locked.

The safety population consists of all participants who received at least one dose of study medication.

The primary efficacy variable, difference in change from baseline to week 52 in residual insulin secretion measured by stimulated C-peptide 2 hour area under the curve profile measured by MMTT, will be estimated by ANCOVA adjusting for differences between treatment groups in stimulated C-peptide 2 hour area under the curve at baseline. The secondary efficacy variables (difference in change in insulin need, time in glucose range, time in hypoglycaemia and HbA1c) will also be estimated by ANCOVA adjusting for difference in the evaluated variable at baseline. No interim analyses are planned in the study.

Regarding continuous exploratory variables, the difference between the two groups for normally distributed variables will be analysed using ANCOVA adjusting for baseline value and for not normally distributed variables using Fisher’s non-parametric permutation test. Dichotomous variables will be analysed using Fisher’s exact test and ordered categorical variables using Mantel-Haenszel Chi-square trend test. Statistiska Konsultgruppen, Gothenburg, Sweden, has been consulted for statistical methods.

### Sample size calculation

In a previous study of 191 individuals with type 1 diabetes followed from diagnosis and year onwards, stimulated c-peptide decreased from 0.70 to 0.46 nmol/L.^6^ The observed SD for c-peptide was 0.34 nmol/L. In two other studies, the SD has been suggested to be 0.25 nmol/L.^22 23^

Using an SD of 0.34 nmol/L, our power calculations indicate that 114 participants would be needed to detect a 25% reduction in c-peptide decline (0.18 nmol/L difference), with two-sided t-test with a power of >80% and significance level of 0.05. If the SD is 0.25 nmol/L, we will also have power to detect a treatment difference of 0.14 nmol/L. We have, therefore, planned to include 114 participants, with an assumed dropout rate of 10%, 127 participants are required.

### Patient and public involvement

People with diabetes and patient organisations were involved in overall discussions of the design of the study for comments regarding relevance of the study aim and outcome measures.

## Ethics and dissemination

The study protocol complies with the Declaration of Helsinki. We obtained approval for this study from the Swedish Ethical Review Authority (Dnr 2020–05098) and the Swedish Medical Products Agency (Dnr 5.1-2021-105808). Protocol changes are submitted as amendments to the Swedish Ethical Review Authority and the Swedish Medical Products Agency. Verbal and written informed consent for participation in the study will be obtained from all participating individuals (see online supplemental file for consent form). The results of this study will be submitted for publication in international peer-reviewed journals and the key findings will be presented at international scientific conferences.

## Discussion

Although IL-17 has been proposed as a key mechanism for destruction of beta cells in type 1 diabetes during the past two decades, this study is the first pivotal trial performed to investigate potential beneficial effects of inhibiting IL-17 in people with newly diagnosed type 1 diabetes.

The IL-17 family consists of six structurally related cytokines, IL-17A-F.^24^ The members of the IL-17 family adopt a monomer fold typical of cystine knot growth factors.^25^ IL-17 is the signature cytokine of Th17 cells, which is activated by the IL-12 family cytokine, IL-23. IL-17A is most similar to IL-17F in both cellular origin and function (~60%) and the genes encoding them are proximally located on chromosome 6. These two cytokines are co-expressed from linked genes and are typically co-produced by Th17 cells.^26^ IL-17 is also expressed by other adaptive and immune cells, including CD8+T cells, natural killer T cells and innate lymphoid cells. IL-17 is known for its ability to initiate a potent inflammatory response.^24^,^26^ The study medication, ixekizumab, is a monoclonal IgG4 antibody binding with high affinity to IL-17A.^27^

Several studies suggest that IL-17 plays a key role in the destruction of beta cells and the development of type 1 diabetes.^7^,^15^ In non-obese diabetic (NOD) mice, IL-17A/F expression is increased within the islets and correlates with diabetes progression.^8^ In humans, upregulated Th-17 immunity has been detected in peripheral T cells from children with type 1 diabetes,^12^ and adults with long-standing type 1 diabetes also show higher levels of Th17 cells compared with age- and sex-matched controls.^10^ Pharmacological inhibition of the IL-12/23 pathway in a small cohort of adults with new-onset type 1 diabetes reduced IL-17A-producing T cells, supporting a pathogenic role for this pathway.^13^,^15^ In a randomised, placebo-controlled trial in new-onset type 1 diabetes, inhibition of IL-12/23 preserved C-peptide, and mechanistic analyses implicated that the IL-17 pathway was involved.^9^ Complementing these data, a recent population-based study in Denmark evaluated molecular biomarkers and their temporal association with incident diabetes in Danish blood donors. The study identified that IL-17A/F was a type 1 diabetes-specific temporal biomarker.^11^ Together, these findings strengthen the concept that IL-17 plays a role in the development of type 1 diabetes. However, animal experiments in NOD mice indicate that IL-17 secreting Th-17 cells may convert to IFN-γ secreting cells. This plasticity may limit the efficacy of IL-17-targeted therapies, as it could potentially accelerate type 1 diabetes in humans.^28^ While the relevance of this is not known in humans, it means a potential risk of selectively blocking IL-17 without acting on a broader level on Th1 response.

In the pancreas of newly diagnosed people with type 1 diabetes, few islets show peri-insulitis, and in these lymphocytic lesions, a large proportion is tissue-resident memory (T_RM_) cells, accounting for approximately 40% of the total number of CD8+T cells per inflamed islet—a proportion similar to what is seen in skin lesions of psoriasis.^29^ In psoriasis, CD8+T_RM_ cells from skin lesions predominantly generate IL-17 responses that promote local inflammation.^30^ Several studies also show direct negative effects of IL-17 on beta cells, ie, increased apoptosis and upregulated expression of stress response genes and proinflammatory chemokines.^31 32^ Inhibition of IL-17 in individuals with psoriasis has shown marked positive effects.^33^

Several earlier studies have been performed with the aim of preserving beta cell function. These studies have targeted different immunological processes with various effects on the change in residual insulin secretion.^2334^,^37^ A difference with the current study medication, the anti-IL-17 (ixekizumab), is that it efficiently affects IL-17.

If the IL-17 signalling pathway is effectively blocked, it is possible that the destruction of beta cells in the pancreas could be halted. Preservation of beta cell function would reduce the dependency of external insulin therapy and minimise the risk of hypoglycaemia, diabetic ketoacidosis and long-term complications. The glycaemic control would likely improve, as well as quality of life. Preservation of endogenous insulin production is viewed as one of the focus areas in the development of new treatment options for people with type 1 diabetes.^38^

Strengths of the study are the randomised, double-blind, placebo-controlled study design. Another strength is the secondary outcomes that will offer information regarding insulin doses and glucose metrics measured by masked continuous glucose monitoring. Additionally, the extension phase will give further knowledge whether a potential beneficial effect is sustained over time. Limitations include the lack of data on how children respond to the treatment, and the follow-up period is relatively short at 52 weeks during ixekizumab/placebo treatment.

In summary, IL-17 is a novel pathway that has not been previously evaluated in a pivotal trial for treatment in newly diagnosed individuals with type 1 diabetes. If it preserves endogenous insulin production, it could be essential for improving glucose control, simplifying treatment regimens and reducing risk of complications in persons with type 1 diabetes. Further, it may be essential also for delaying and preventing the onset of type 1 diabetes.

## Supplementary material

10.1136/bmjopen-2025-103486 online supplemental file 1

10.1136/bmjopen-2025-103486 online supplemental file 2
