# Supplementary material for: Design and methods of the Ixekizumab Diabetes Intervention Trial (I-DIT): protocol for a phase 2, randomised, multicentre, placebo-controlled, double-blind trial of anti-interleukin 17 as a treatment option for adults with new-onset type 1 diabetes
Source: BMJ Open. 2025 Nov 12;15(11):e103486. doi: 10.1136/bmjopen-2025-103486 (PMC12612750; doi:10.1136/bmjopen-2025-103486)
Supplement: online supplemental file 1 [file bmjopen-15-11-s001.docx]

**Information till forskningspersonerna**

**Effekten av IL-17-hämning hos nydiagnostiserade personer med typ 1 diabetes**

**Härmed tillfrågas du som nyligen insjuknat i typ 1 diabetes och påbörjat insulinbehandling under de senaste 100 dagarna om ditt intresse av att delta i följande studie*:***

**Vad är det för projekt och varför vill ni att jag ska delta?**

Typ 1 diabetes är en immunologisk sjukdom som uppstår på grund av att de insulinproducerande betacellerna i bukspottskörteln angrips av kroppens egna immunsystem. Förlusten av betacellerna sker långsamt över flera år. Typ 1 diabetes leder till att kroppen inte kan producera tillräckligt med insulin och man blir beroende av livslång insulinbehandling. Det finns vetenskapligt underlag för att det finns fungerande insulinproducerande betaceller kvar efter diagnos.

Syftet med denna studie är att rädda de kvarvarande insulinproducerande cellerna (betaceller) och minska inflammationen i bukspottskörteln med ett studieläkemedel.

Man har sett att det finns en del likheter mellan typ 1 diabetes och hudsjukdomen psoriasis. När man tagit vävnadsprov från bukspottskörtel och psoriasis-fläckar har man sett att det finns liknande inflammatoriska celler som utsöndrar proteinet interleukin-17 (IL-17).

Studieläkemedlet, Ixekizumab, tillhör en läkemedelsgrupp som kallas interleukin-(IL)-hämmare och är godkänt att använda för sjukdomen psoriasis. Läkemedlet ges som en injektion under huden. Ixekizumab binder till proteinet IL-17 och blockerar dess effekt.

Studier har visat att aktivering av IL-17 också är ett kännetecken för sjukdomsuppkomsten av typ 1 diabetes. Vi är därför intresserade av att studera effekten av Ixekixumab hos individer nyligen diagnosticerade med typ 1 diabetes.

I denna studie kommer 127 deltagare att slumpas till två olika grupper. En grupp kommer att få studieläkemedlet Ixekizumab och den andra gruppen får placebo i tillägg till sin sedvanliga insulinbehandling. Studien är blindad, vilket innebär att varken du som studiedeltagare eller vi i forskargruppen vet vilken behandlingsgrupp du hamnat i. Alla studiedeltagare kommer att följas i 1 år. Cirka 1 och 3 år efter att studiebehandlingen har avslutats kommer du också att lämna blodprover hos en sjuksköterska under ett besök för att vi ska kunna utvärdera om man ser långtidseffekter av behandlingen.

*Forskningshuvudman för projektet är Göteborgs Universitet.*

**Förfrågan om deltagande**

I studien tillfrågas och inkluderas personer som är 18-45 år som nyligen insjuknat i typ 1 diabetes och påbörjat insulinbehandling de senaste 100 dagarna.

**Hur går studien till?**

Du som deltar i studien kommer slumpas till att antingen få läkemedlet Ixekizumab eller placebo. Ixekizumab är en förfylld spruta som tas via huden. Den första veckan kommer du att ta 2 injektioner, och därefter en injektion vecka 2, 4, 6, 8, 10 och 12. Därefter tar du läkemedlet var 4:e vecka under en total behandlingstid på 12 månader. Via telefon följs du upp av en sjuksköterska dag 1, dag 3 och en vecka efter du tagit läkemedlet. Alla deltagare kommer att träffa sjuksköterska efter vecka 2, 4, 13, 26, 39 och 52 för blodprovstagning, genomgång av eventuella biverkningar och sedvanlig rådgivning om behandling.

***Kontakt 1***

Den första skriftliga och muntliga informationen om studien får du antingen via ett besök på den klinik som kontaktat dig eller via brev och telefon. Du får möjlighet att läsa igenom patientinformationen i lugn och ro och ställa frågor innan du bestämmer dig för om du vill delta. Om du ger skriftligt och muntligt medgivande att delta i studien kommer vi att gå igenom villkoren för om du kan delta i studien.

***Screeningsbesök 1***

Ifall du väljer att delta i studien och har givit muntligt och skriftligt samtycke till detta kommer studien att börja med ett screeningbesök. Syftet är att ta reda på om du uppfyller kraven för att delta i forskningsstudien. Det kommer då att tas blodprover på dig (t.ex. prover avseende blodsockerkontroll, riskfaktorer för följdsjukdomar till diabetes, leverprover, blodvärde, hepatit, HIV och tuberkulos). Efter detta besök kommer du också att genomföra en slätröntgen (lungröntgen) av lungorna för att se att det inte finns förändringar som vid tuberkulos. Om du inte kan vara med i studien kommer du att få veta varför. Under detta besök kommer vi också sätta en blindad glukosmonitorering på dig, som kommer att mäta blodsockret, under 2 veckor. Om du kan delta i studien blir du ombedd att återkomma till kliniken för besök 2.

***Studieläkemedel, besök 2***

Vid besök 2 kommer du att slumpas till att få studieläkemedlet eller till placebo. Vid detta besök kontrolleras också vikt, blodtryck och HbA1c (långtidssocker) och insulindoser.

**Studieschema**

***Besök och telefonkontakt under studiens gång***

Efter besök 1 (screeningbesöket) och besök 2 (där du får studieläkemedlet) kommer du att återkomma till kliniken för ytterligare 6 besök för blodprovstagning och kliniska undersökningar under 1 år.

Under ett besök, cirka 1 och 3 år efter att studiebehandlingen har avslutats kommer du också att lämna blodprover hos en sjuksköterska för uppföljning.

***Besök till sjuksköterska under studien***

Du kommer att träffa sjuksköterska vid studiestart, vecka 2, 4, 13, 26, 39 och 52. Vid samtliga besök kommer HbA1c (långtidsblodsocker), blodsockernivå och insulindoser och diabetesmediciner att registreras samt vikt och blodtryck kontrolleras. Vid 4 tillfällen kommer du att bära en glukosmonitorering (blodsockermätare) under 2 veckor.

Vi är också intresserade av att ta reda på hur mycket insulin din bukspottskörtel producerar. Det kommer vi att mäta genom ett så kallat Mixed Meal Tolerance Test (MMTT), vilket innebär att vi tar ett blodprov efter att du fått dricka en ”blandad måltid” som innehåller protein, kolhydrater och fett. Blodprover tas -10 min och 0 min (innan du dricker måltiden) och därefter 15, 30, 60, 90 och 120 min. MMTT görs vid följande besök: screening, besök 2 (när du slumpas till behandling), samt vecka 4, 26, 52 och 1 och 3 år efter att studiebehandlingen har avslutats.

Vi vill också veta hur dina insulindoser förändras under studiens gång, du kommer därför fylla i ett formulär och registrera dina insulindoser under 5 dagar vid besök 2 (när du slumpas till behandling), vecka 4, 26, 52 och 1 och 3 år efter att studiebehandlingen har avslutats.

Vi kommer tillfråga om oönskade händelser, ifall du t.ex. haft någon allvarlig hypoglykemi (lågt blodsocker) eller syraförgiftning (ketoacidos, högt blodsocker). Under studien kommer du vid 3 tillfällen också få fylla i ett par enkäter om behandlingstillfredsställelse, mående och fysisk aktivitet.

***Läkarbesök***

Du kommer att träffa läkare i samband med att kriterierna gås igenom för att se om du passar för studien. Du kommer även att träffa läkare fortlöpande under studien under dina planerade besök. Vid klinisk indikation kommer ytterligare läkarbesök att ske.

**Blodprover**

Den totala mängden blod som tas vid varje besök är 15-100 ml, vilket motsvarar cirka 1-7 matskedar blod.

**Möjliga följder och risker med att delta i studien**

Studieläkemedlet Ixekizumab tas genom injektion under huden. Det kan innebära ett visst obehag och risk för blåmärke på insticksstället. Under studiens gång kan du behöva ändra din insulinbehandling och det kan medföra ökade risker för variationer i blodsockret (höga och låga blodsockervärden), men med stöttning av sjuksköterska och läkare bedömer vi att riskerna är små avseende detta. Registrering av oönskade medicinska händelser samt biverkningar kommer att ske kontinuerligt under studien.

Vid tandbehandlingar (inklusive tandblekning) med risk för blödning eller infektion kan eventuellt profylaktisk antibiotikabehandling behöva sättas in. Det är bra om du tar upp det med din tandläkare.  Ifall det uppkommer under studiens gång att du ska genomgå ett kirurgiskt ingrepp ska du kontakta den läkare som är ansvarig på din studieenhet för att ta ställning till om studieläkemedel ska sättas ut inför kirurgi. Ta isåfall kontakt så snart kirurgi är planerad.

Behandling med Ixekizumab kan förknippas med ökad infektionsfrekvens. Vid infektion ska du ta kontakt med studieteamet på din vårdenhet. Du kommer att övervakas noga och behandlingen sätts ut om du inte svarar på standardbehandling eller om en infektion blir allvarlig eller kräver sjukhusvård.

Du kommer att få ett mindre kort av oss som du alltid skall ha med dig (exempelvis i plånboken) där kontaktuppgifter finns för läkare i händelse av att man i en akut situation vill veta om du erhållit det aktiva studieläkemedlet eller placebo. Om du är i behov av läkarvård bör du lämna över ditt patientkort till sjukvårdspersonalen och tala om att du deltar i en klinisk studie.

För inkludering i studien behöver du genomföra en lungröntgen. Du kommer att få en liten stråldos under undersökningen. Det är dock en låg dos, och den dosen fås normalt per år från naturliga källor för oss som bor i Sverige. Du mår som vanligt efter lungröntgen.

**Graviditet**

Du får inte vara gravid eller planera graviditet när du börjar i studien. Du får inte heller planera att bli gravid inom 4 månader efter att du fått sista behandlingstillfället med studieläkemedlet. Om du är kvinna eller man måste du under hela studieperioden använda säkra preventivmetoder vid sexuella kontakter.

**Finns det några fördelar med att delta i studien**

Du kommer att få ökade kunskaper om diabetes samtidigt som du bidrar till ökad kunskap om sjukdomsutvecklingen vid typ 1 diabetes. Om du får aktivt studieläkemedel och behandlingen fungerar kommer din diabetes att kunna hanteras på ett enklare sätt på grund av att insulinproducerande betaceller bevaras.

Eftersom viss egen insulinproduktion ofta finns i flera år efter diagnos av diabetes kan du som deltar eventuellt ha nytta av behandlingen i framtiden om den visar gynnsamma effekter. Du kommer att följas upp noggrant under studien med både klinikbesök och telefonsamtal.

**Vad händer med mina uppgifter?**

Projektet kommer att samla in och registrera information om dig.

En person med specialkompetens (s.k. monitor) kommer besöka din vårdenhet och gå igenom studiedata och journaldata som är kopplade till studien. Denna person har till uppgift att säkerställa att studiedata och undersökningar i studien utförs på korrekt sätt enligt den forskningsplan som har godkänts för studien av Etikprövningsmyndigheten. En monitor ska finnas för den aktuella typen av studier enligt regelverk för dessa studier för att bidra till att den utförs på ett så korrekt sätt som möjligt. Monitorn gör detta på uppdrag av den organisation som är huvudansvarig för studien, i detta fall Göteborgs Universitet. Monitorn kommer därför ha tillgång till dina studiedata och kommer lyda under sedvanlig sekretess som råder för personer som har tillgång till journaldata för patienter på din klinik. En s.k. kodnyckel kommer finnas på din klinik vilket innebär att inga personer utöver personal på din klinik och monitorn kommer ha tillgång till ditt namn och personnummer när data analyseras vidare av de ansvariga för studien.

Ansvarig för dina personuppgifter är Göteborgs Universitet. Enligt EU:s dataskyddsförordning har du rätt att kostnadsfritt få ta del av de uppgifter om dig som hanteras i studien, och vid behov få eventuella fel rättade. Du kan också begära att uppgifter om dig raderas samt att behandlingen av dina personuppgifter begränsas. Om du vill ta del av uppgifterna ska du kontakta ansvarig forskare på site _______ på telefon____. Dataskyddsombud är Göteborgs Universitet, och kan nås på e-post [dataskyddsombud@gu.se](mailto:dataskyddsombud@gu.se).

Du har möjlighet att lämna klagomål till Integritetsskyddsmyndigheten om du anser att behandlingen av personuppgifter strider mot dataskyddsförordningen (GDPR).

Ansvarig läkare är ansvarig för hantering av studiedata. Insamlade studiedata lagras, bearbetas och sammanställs av forskare både manuellt och med datorteknik. Sponsorn kan överföra din kodade studieinformation till medarbetare, forskningspartners eller andra som analyserar data i anslutning till studien eller framtida eller relaterade studier. Syftet med att genomföra studien är forskning och utveckling inom diabetesvården som beskrivits i denna information.

**Vad händer med mina prover?**

De prover som tas i studien förvaras kodade i en så kallad biobank. Biobankens namn är Biobank Väst 890 och den finns vid Göteborg. Huvudman (ansvarig) för biobanken är Västra Götalandsregionen. Prov hanteras i enlighet med den svenska biobankslagen (2023:38).

Dina blodprover kan komma att skickas till olika internationella laboratorier för specifika analyser avseende diabetes, t.ex. analyser av olika ämnen som kan ha betydelse för att förstöra de insulinproducerande betacellerna i bukspottskörteln. Dessa prover kommer att skickas anonymiserade (utan ditt namn eller personnummer) och gruppvis samt förstöras efter analys.

Du har rätt att säga nej till att proverna sparas. Om du samtycker till att proverna sparas har du rätt att senare ta tillbaka (ångra) det samtycket. Dina prover kommer i så fall att kastas eller avidentifieras. Om du vill ångra ett samtycke ska du kontakta Dr. _____ på telefon _____.

Proverna får bara användas på det sätt som du har gett samtycke till. Om det skulle tillkomma forskning som ännu inte är planerad, kommer Etikprövningsmyndigheten att besluta om du ska tillfrågas på nytt.

**Hur får jag information om resultatet av studien?**

Resultaten från studien kan komma att publiceras i vetenskapliga tidskrifter. Resultaten redovisas för hela grupper och röjer därmed inte den enskilda individens personuppgifter

**Försäkring och ersättning**

Att vara med i studien medför inga kostnader för dig. Liksom inom sjukvården i övrigt omfattas du av Patientförsäkringen. Ersättning utgår i form av 300 kr per besök och reseersättning som utbetalas efter att studien avslutats. Detta för att kompensera för att du tar dig tid att vara med i studien. Du får också ersättning för resekostnader.

**Deltagandet är frivilligt**

Ditt deltagande är frivilligt och du kan när som helst välja att avbryta deltagandet. Om du väljer att inte delta eller vill avbryta ditt deltagande behöver du inte uppge varför, och det kommer inte heller att påverka din framtida vård eller behandling.

Om du vill avbryta ditt deltagande ska du kontakta den ansvariga för studien (se nedan).

**Ansvariga för studien**

| Studiesköterska: | Telefon: |
| --- | --- |
| Ansvarig läkare: | Telefon: |

|  |  |  |  |  |
| --- | --- | --- | --- | --- |
|  | Patientens initialer: |  | Screeningnummer: |  |
|  |  |  |  |  |

**Patientens samtycke**

Jag har muntligen informerats om ovanstående studie och läst bifogad skriftlig information. Jag har fått tillfälle att ställa frågor och fått eventuella frågor besvarade.

Jag samtycker till:

- Jag samtycker till att delta i studien ” Effekten av IL-17-hämning hos nydiagnostiserade personer med typ 1 diabetes”
- Jag samtycker till att uppgifter om mig behandlas på det sätt som beskrivs i forskningspersonsinformationen.
- Jag samtycker till att mina prover sparas i en biobank på det sätt som beskrivs i forskningspersonsinformationen.

Jag känner till att mitt deltagande är helt frivilligt. Jag är medveten om att jag när som helst och utan närmare förklaring kan avbryta mitt deltagande utan att detta påverkar mitt framtida omhändertagande.

|  |  |  |
| --- | --- | --- |
| Ort |  | Patientens underskrift |
|  |  |  |
| Datum |  | Namnförtydligande |
|  |  |  |
| Information lämnad av: |  |  |
|  |  |  |
| Ort |  | Namnteckning |
|  |  |  |
| Datum |  | Namnförtydligande |
|  |  | Titel |

**En kopia av denna signerade patientinformation ges till patienten**
