# Supplementary material for: Design and methods of the Ixekizumab Diabetes Intervention Trial (I-DIT): protocol for a phase 2, randomised, multicentre, placebo-controlled, double-blind trial of anti-interleukin 17 as a treatment option for adults with new-onset type 1 diabetes
Source: BMJ Open. 2025 Nov 12;15(11):e103486. doi: 10.1136/bmjopen-2025-103486 (PMC12612750; doi:10.1136/bmjopen-2025-103486)
Supplement: online supplemental file 2 [file bmjopen-15-11-s002.docx]

# **Sites**

**Västra Götalandsregionen:**

NU-Hospital Group, Trollhättan/Uddevalla

Sahlgrenska University Hospital/Sahlgrenska, Göteborg

Sahlgrenska University Hospital/Östra, Göteborg

Södra Älvsborgs Hospital, Borås

Skaraborgs Hospital, Skövde

**Region Stockholm:**

Centrum för Diabetes, Stockholm

Södersjukhuset AB, Stockholm

**Region Uppsala:**

Clinical Trial Center, Uppsala

**Region Skåne:**

Klinisk Prövningsenhet, Lund

Central Hospital Kristianstad, Kristianstad

**Region Värmland**

Central Hospital Karlstad, Karlstad

**Region Halland:**

Varberg Hospital, Varberg

**Region Jönköpings län**

Ryhov County Hospital, Jönköping

**Region Östergötland:**

Linköping University Hospital, Linköping

Vrinnevi Hospital Norrköping, Norrköping

**Region Örebro län:**

Örebro University Hospital, Örebro

**Region Dalarna:**

Falu Hospital, Falun

# **Mixed Meal Tolerance Test (MMTT)**

The mixed meal will consist of a standard liquid meal Fresubin® (or equivalent). The product and flavour selected and used at the first visit will be recorded on the CRF and the participant will be required to consume the same product at all subsequent visits. The subjects have an overnight fast >10 hours before the test. At starting time 0, the nutrient will be consumed over

5 minutes.

The MMTT assesses the participant’s insulin production capacity. The MMTT will be performed at screening, randomisation, week 4, week 26, week 52, week 104 and week 208 after starting the study medication. The test must be performed between 7-10 am. From midnight, the evening preceding the test, the subjects will be permitted to drink only water but not eat. Vigorous exercise, coffee, tea, caffeine-containing drinks and cigarettes are not allowed within the 10 hours prior to the test. If using insulin pump, the basal insulin must be given as usual. The participant shall take his/her regular injection with basal insulin day/night before but not in the morning before MMTT. Rapid acting insulin analogues may be taken as boluses via insulin pump or as injection up to 4 hours before the test. Participants with an advanced insulin pump using auto-correction of insulin doses shall stop the auto-correction function at the latest 2 hours before MMTT-test.

If for safety reasons, subjects need to take rapid acting insulin analogue (as injection or as bolus via insulin pump) due to hyperglycaemia or Dextrose 0.3 g/kg bodyweight due to hypoglycaemia as within 4 hours before the test, the visit will be rescheduled. The MMTT will not be performed if the fasting rules are not followed, the test will then be rescheduled. The MMTT will only be performed if the fasting plasma glucose is between 4-12 mmol/L on the participant’s own glucose meter in the morning of the test. If the plasma glucose is lower than 4 mmol/l or higher than 12 mmol/l, the test will be rescheduled.

Patients must continue to have ongoing basal insulin dose during the last 6 hours before the

MMTT. If the conditions above are not fulfilled the MMTT will be rescheduled and performed within 7 days.

1. The MMTT procedure starts between 7-10am after 10 hours fasting.
2. Patients have to be weighed, and the weight registered in the eCRF.
3. The amount of Fresubin® (or equivalent) should be calculated, 6ml/kg, maximum 360ml.
4. Insert a cannula in a large antecubital vein, local anesthetic cream is acceptable.
5. Obtain baseline blood samples
   1. First blood sample 10 minutes before the liquid meal (Time -10)
   2. Second blood samples should be taken from the cannula just prior to drinking the standardized liquid meal –this is the 0-minute sample. Timer should be started at 0 minutes.
6. Meal consumption: Start the clock at the beginning of the drink. The meal must be completely swallowed within 5 minutes.
7. Post-meal blood samples: Samples are taken at 15, 30, 60, 90 and 120 minutes (+/- 5 minutes) after ingestion of meal. Timer should be turned on at 0 minutes.
8. Sampling protocol:

Time (minutes) C-peptide/plasma glucose

-10 X

0 X

15 X

30 X

60 X

1. X
2. X
3. Clogged line, missed sample and other deviations from the protocol must be recorded.

# **Examples of contraceptive methods**

Male subjects agree to use a reliable method of birth control during the study.

Female subjects:

Participants of childbearing age or childbearing potential who are sexually active who

test negative for pregnancy must be counseled and agree to use either 1 highly

effective method of contraception or 2 acceptable methods of contraception combined

for the duration of the study and for at least 12 weeks following the last dose of study

drug or remain abstinent during the study and for at least 12 weeks following the last

dose of study drug.

If the highly effective contraceptive methods are contraindicated or strictly declined by

patient, acceptable birth control methods may be considered. These may include combination of both of the following methods:

- Male or female condom with spermicide
- Cap, diaphragm, or sponge with spermicide

1. Highly effective methods of contraception (use 1 form):

combined oral contraceptive pill and mini-pill

- NuvaRing®
- implantable contraceptives
- injectable contraceptives (such as Depo-Provera®)
- intrauterine device (such as Mirena® and ParaGard®)
- contraceptive patch—ONLY women 90 kg or <198 pounds
- abstinence from sex
- vasectomy—for men in clinical studies

2. Effective methods of contraception (use 2 forms combined)

- male condom with spermicide
- female condom with spermicide
- diaphragm with spermicide
- cervical sponge
- cervical cap with spermicide

Females who are not of childbearing potential include those who have undergone or

who have:

- female sterilization
- hysterectomy
- menopause
- Müllerian agenesis (Mayer–Rokitansky–Küster–Hauser syndrome [also
- referred to ascongenital absence of the uterus and vagina])
