# Supplementary material for: Design and methods of the Ixekizumab Diabetes Intervention Trial (I-DIT): protocol for a phase 2, randomised, multicentre, placebo-controlled, double-blind trial of anti-interleukin 17 as a treatment option for adults with new-onset type 1 diabetes
Source: BMJ Open. 2025 Nov 12;15(11):e103486. doi: 10.1136/bmjopen-2025-103486 (PMC12612750; doi:10.1136/bmjopen-2025-103486)
Supplement: Supplementary file 3 [file bmjopen-2025-103486.reviewer_comments.pdf]

PEER REVIEW HISTORY

BMJ Open publishes all reviews undertaken for accepted manuscripts. Reviewers are asked to complete a checklist review form and are provided with free text boxes to elaborate on their assessment. These free text comments are reproduced below.

ARTICLE DETAILS

Title (Provisional)

Design and methods of the Ixekizumab Diabetes Intervention Trial (I-DIT): protocol for a phase 2, randomized, multicentre, placebo-controlled, double-blind trial of anti-interleukin 17 as a treatment option for adults with new-onset type 1 diabetes

Authors

S Ahmadi, Shilan; Korsgren, Olle; Jansson, Per-Anders; Lind, Marcus

---

VERSION 1 - REVIEW

**Reviewer**	**1**
**Name**	**Marwaha, Ashish**
**Affiliation**	**University of Calgary, Department of Medical Genetics**
**Date**	**03-Sep-2025**
**COI**	**None**

Protocol paper and the methods seem standard for clinical trials in the field, Few suggestions before publication:

1. some references are wrongly cited in the text (eg 9 and 11 seem to be swapped- please double check all the references are accurate for what you are describing in the text)

2.would recommend that it is not claimed that this is the first study of the IL-17 pathway in T1D (it is accurate to say it will be the first study to complete recruitment that looked at IL17A/F inhibition specifically in T1D) . The IL17 pathway has been examined previously in T1D via clinical trial and to say it hasn't is inaccurate given that a lot of previous studies have examined IL-23 inhibitor ustekinumab and specifically looked at the mechanistic mechanis to identify an IL-17 pathway effect on efficacy. You have cited one of these studies and actually that is the main direct rationale for examining this pathway in humans as the other evidence mentioned is weaker. You may want to point out that in the mechanistic analyses of this trial in nature medicine there was a population of IL-17 secreting cells that was identified as a biomarker of efficacy. Also it is weakness of the proposed trial design that samples are not

being run for any mechanistic analyses to examine the effect of this drug on IL-17 cell enumeration or function (eg Th17) incase there is not a complete response in all participants we will not know why. The study referring to all this is cited but should be expanded on for the rationale:

Ustekinumab for type 1 diabetes in adolescents: a multicenter, double-blind, randomized phase 2 trial.

Tatovic D, Marwaha A, Taylor P, Hanna SJ, Carter K, Cheung WY, Luzio S, Dunseath G, Hutchings HA, Holland G, Hiles S, Fegan G, Williams E, Yang JHM, Domingo-Vila C, Pollock E, Wadud M, Ward-Hartstonge K, Marques-Jones S, Bowen-Morris J, Stenson R, Levings MK, Gregory JW, Tree TIM, Dayan C; USTEKID Study Group.

Nat Med. 2024 Sep;30(9):2657-2666. doi: 10.1038/s41591-024-03115-2. Epub 2024 Jul 30.

PMID: 39079992 Free PMC article. Clinical Trial.

3.Several papers are missing from the rationale that were crucial is establishing the rationale for this trial and should be cited or mentioned in addition to the selected ones from the literature presented and briefly described.:

A phase 1b open-label dose-finding study of ustekinumab in young adults with type 1 diabetes.

Marwaha AK, Chow S, Pesenacker AM, Cook L, Sun A, Long SA, Yang JHM, Ward-Hartstonge KA, Williams E, Domingo-Vila C, Halani K, Harris KM, Tree TIM, Levings MK, Elliott T, Tan R, Dutz JP.

Immunother Adv. 2021 Nov 13;2(1):ltab022. doi: 10.1093/immadv/ltab022. eCollection 2022.

PMID: 35072168

Targeting the IL-17/IFN-γ axis as a potential new clinical therapy for type 1 diabetes.

Marwaha AK, Tan S, Dutz JP.

Clin Immunol. 2014 Sep;154(1):84-9. doi: 10.1016/j.clim.2014.06.006. Epub 2014 Jun 16.

PMID: 24947953 Review.

Cutting edge: Increased IL-17-secreting T cells in children with new-onset type 1 diabetes.

Marwaha AK, Crome SQ, Panagiotopoulos C, Berg KB, Qin H, Ouyang Q, Xu L, Priatel JJ, Levings MK, Tan R.

J Immunol. 2010 Oct 1;185(7):3814-8. doi: 10.4049/jimmunol.1001860. Epub 2010 Sep 1.

PMID: 20810982

4. mouse work has shown that IL-17 secreting th17 cells in NOD mice models may convert to IFNgamma secreting cells and this could be a disadvantage of targeting IL-17 alone vs IFNg and a major safety concern potentially with this approach, this should be discussed as a potential complication of accelerating T1D in humans.

Polarized Th17 cells from the BDC2.5 NOD mouse accelerate T1D in NOD SCID recipient only after differentiating

into a Th1 phenotype.

[19] D. Bending, et al., Highly purified Th17 cells from BDC2.5NOD

mice convert into Th1-like cells in NOD/SCID recipient mice, J.

Clin. Invest. 119 (3) (2009) 565–572.

[20] N. Martin-Orozco, et al., Th17 cells promote pancreatic

inflammation but only induce diabetes efficiently in lympho penic hosts after conversion into Th1 cells, Eur. J. Immunol. 39

(1) (2009) 216–224.

5. The authors suggest Izekizumab targets IL17A and IL17F both and reference a statpearls article. On looking up this article it mentions only IL17A

Ixekizumab is a humanized monoclonal antibody (IgG) that selectively binds to interleukin-17A (IL-17A), thereby preventing it from binding to the IL-17 receptor. This attenuates an inflammatory response mediated by interleukin-17A.[12]

The authors should correct this misrepresentation of the mechanism of action of the drug as there are other IL-17A and F inhibitors available on the market and so this may be an important difference in T1D efficacy. If the authors have primary evidence of IL17F inhibition by Izekinumab that needs to be cited directly.

VERSION 1 - AUTHOR RESPONSE

Reviewer: 1
Dr. Ashish Marwaha, University of Calgary

Comments to the Author:
Protocol paper and the methods seem standard for clinical trials in the field. Few suggestions before publication:

*** Some references are wrongly cited in the text (eg 9 and 11 seem to be swapped- please double check all the references are accurate for what you are describing in the text)**

Response: Thank you for the comment. The references have been changed accordingly.

*** Would recommend that it is not claimed that this is the first study of the IL-17 pathway in T1D (it is accurate to say it will be the first study to complete recruitment that looked at IL17A/F inhibition specifically in T1D) . The IL17 pathway has been examined previously in T1D via clinical trial and to say it hasn't is inaccurate given that a lot of previous studies have examined IL-23 inhibitor ustekinumab and specifically looked at the mechanistic mechanism to identify an IL-17 pathway effect on efficacy. You have cited one of these studies and actually that is the main direct rationale for examining this pathway in humans as the other evidence mentioned is weaker. You may want to point out that in the mechanistic analyses of this trial in nature medicine there was a population of IL-17 secreting cells that was identified as a biomarker of efficacy. Also, it is weakness of the proposed trial design that samples are not being run for any mechanistic analyses to examine the effect of this drug on IL-17 cell enumeration or function (eg Th17) in case there is not a complete response in all participants we will not know why. The study referring to all this is cited but should be expanded on for the rationale:**

Ustekinumab for type 1 diabetes in adolescents: a multicenter, double-blind, randomized phase 2 trial.
Tatovic D, Marwaha A, Taylor P, Hanna SJ, Carter K, Cheung WY, Luzio S, Dunseath G, Hutchings HA, Holland G, Hiles S, Fegan G, Williams E, Yang JHM, Domingo-Vila C, Pollock E, Wadud M, Ward-Hartstonge K, Marques-Jones S, Bowen-Morris J, Stenson R, Levings MK, Gregory JW, Tree TIM, Dayan C; USTEKID Study Group.
Nat Med. 2024 Sep;30(9):2657-2666. doi: 10.1038/s41591-024-03115-2. Epub 2024 Jul 30.
PMID: 39079992 Free PMC article. Clinical Trial.

Response: Thank you for the comment. We changed the sentence where it says “first”. We have also pointed out that it was in the mechanistic analysis of Ustekizumab-trial IL-17A were identified as a biomarker of efficacy. Regarding mechanistic analysis in our study, biobank samples (blood, serum and whole blood) are collected for all participants and in a subsample of participants also peripheral mononuclear cells as written under “lab, monitor and study drug delivery”-section.

3. Several papers are missing from the rationale that were crucial is establishing the rationale for this trial and should be cited or mentioned in addition to the selected ones from the literature presented and briefly described.:

A phase 1b open-label dose-finding study of ustekinumab in young adults with type 1 diabetes. Marwaha AK, Chow S, Pesenacker AM, Cook L, Sun A, Long SA, Yang JHM, Ward-Hartstonge KA, Williams E, Domingo-Vila C, Halani K, Harris KM, Tree TIM, Levings MK, Elliott T, Tan R, Dutz JP. Immunother Adv. 2021 Nov 13;2(1):ltab022. doi: 10.1093/immadv/ltab022. eCollection 2022. PMID: 35072168

Targeting the IL-17/IFN-γ axis as a potential new clinical therapy for type 1 diabetes.
Marwaha AK, Tan S, Dutz JP.Clin Immunol. 2014 Sep;154(1):84-9. doi:

10.1016/j.clim.2014.06.006. Epub 2014 Jun 16. PMID: 24947953 Review.

Cutting edge: Increased IL-17-secreting T cells in children with new-onset type 1 diabetes. Marwaha AK, Crome SQ, Panagiotopoulos C, Berg KB, Qin H, Ouyang Q, Xu L, Priatel JJ, Levings MK, Tan R. J Immunol. 2010 Oct 1;185(7):3814-8. doi: 10.4049/jimmunol.1001860. Epub 2010 Sep 1.PMID: 20810982

Response: These papers have been added in the manuscript.

4. Mouse work has shown that IL-17 secreting th17 cells in NOD mice models may convert to IFNgamma secreting cells and this could be a disadvantage of targeting IL-17 alone vs IFNg and a major safety concern potentially with this approach, this should be discussed as a potential complication of accelerating T1D in humans.Polarized Th17 cells from the BDC2.5 NOD mouse accelerate T1D in NOD SCID recipient only after differentiating into a Th1 phenotype.

[19] D. Bending, et al., Highly purified Th17 cells from BDC2.5NOD mice convert into Th1-like cells in NOD/SCID recipient mice, J.Clin. Invest. 119 (3) (2009) 565–572.[20] N. Martin-Orozco, et al., Th17 cells promote pancreatic inflammation but only induce diabetes efficiently in lymphopenic hosts after conversion into Th1 cells, Eur. J. Immunol. 39(1) (2009) 216–224.

Response: Thanks, we have added the following section in the discussion:

“However, animal experiments in NOD mice indicate that IL-17 secreting Th-17 cells may convert to IFN-γ secreting cells. This plasticity may limit the efficacy of IL-17-targeted therapies, as it could potentially accelerate type 1 diabetes in humans (28). While the relevance of this is not known in humans it means a potential risk of selectively blocking IL-17 without acting on a broader level on Th1 response.”

5. The authors suggest Izekizumab targets IL17A and IL17F both and reference a statpearls article. On looking up this article it mentions only IL17A Ixekizumab is a humanized monoclonal antibody (IgG) that selectively binds to interleukin-17A (IL-17A), thereby preventing it from binding to the IL-17 receptor. This attenuates an inflammatory response mediated by interleukin-17A.[12]

The authors should correct this misrepresentation of the mechanism of action of the drug as there are other IL-17A and F inhibitors available on the market and so this may be an important difference in T1D efficacy. If the authors have primary evidence of IL17F inhibition by Izekinumab that needs to be cited directly.

Response: Thank you for the comment. The sentence has been changed to IL-17A.
